# Advancements in Neurosurgical Intraoperative Histology

**DOI:** 10.3390/tomography10050054

**Published:** 2024-05-09

**Authors:** Ali A. Mohamed, Emma Sargent, Cooper Williams, Zev Karve, Karthik Nair, Brandon Lucke-Wold

**Affiliations:** 1 Charles E. Schmidt College of Medicine, Florida Atlantic University, Boca Raton, FL 33431, USA; 2 College of Engineering and Computer Science, Florida Atlantic University, Boca Raton, FL 33431, USA; 3 Department of Neurosurgery, University of Florida, Gainesville, FL 32611, USA

**Keywords:** deep neural networks, Raman histology, digital histopathological assessment, frozen sectioning, cytologic preparations

## Abstract

Despite their relatively low incidence globally, central nervous system (CNS) tumors remain amongst the most lethal cancers, with only a few other malignancies surpassing them in 5-year mortality rates. Treatment decisions for brain tumors heavily rely on histopathological analysis, particularly intraoperatively, to guide surgical interventions and optimize patient outcomes. Frozen sectioning has emerged as a vital intraoperative technique, allowing for highly accurate, rapid analysis of tissue samples, although it poses challenges regarding interpretive errors and tissue distortion. Raman histology, based on Raman spectroscopy, has shown great promise in providing label-free, molecular information for accurate intraoperative diagnosis, aiding in tumor resection and the identification of neurodegenerative disease. Techniques including Stimulated Raman Scattering (SRS), Coherent Anti-Stokes Raman Scattering (CARS), Surface-Enhanced Raman Scattering (SERS), and Tip-Enhanced Raman Scattering (TERS) have profoundly enhanced the speed and resolution of Raman imaging. Similarly, Confocal Laser Endomicroscopy (CLE) allows for real-time imaging and the rapid intraoperative histologic evaluation of specimens. While CLE is primarily utilized in gastrointestinal procedures, its application in neurosurgery is promising, particularly in the context of gliomas and meningiomas. This review focuses on discussing the immense progress in intraoperative histology within neurosurgery and provides insight into the impact of these advancements on enhancing patient outcomes.

## 1. Introduction

Cancers of the brain and central nervous system (CNS) are composed of a varied group of pathologies stemming from brain and spinal cord tissue, with the World Health Organization (WHO) classifying over 150 differing brain tumor subtypes [1]. Intracranial and spinal cord malignancies have remained steadfast in ranking among the most fatal cancers, estimating that only esophageal, lung, hepatic, and pancreatic tumors stand ahead of CNS tumor pathology in 5-year mortality rates [2]. While CNS cancers are considered rare when contrasted to other classifications of malignancy (with statistics showing that CNS cancer comprised 1.7% of global cancer cases as compared to 12.2% for cancers of the lung), their place amongst the most fatal cancers has been stout for several years [3,4]. While the 5-year survival rates of brain and CNS cancer are enduringly morbid, mild improvements in the frequency of intracranial malignancy cases have been noted in recent decades, as exemplified by the 0.8% decrease in incidence noted between 2008 and 2017 within North America [5].

The choice of treatment regimen against brain tumors most often anchors on information received from histopathologic analyses of cancerous tissue, an approach that can even be applied intra-operatively to guide the necessary retrieval of additional tissue for biopsy, or improvement in margins with regard to tumor resection and overall morbidity and mortality outcomes [6]. Examples of such growing technique modalities include Raman spectroscopy, frozen sectioning, and confocal laser endomicroscopy [7].

Despite being regarded by some as one of the oldest medical specialties in human history (with some historians arguing for the evidence of neurosurgical techniques performed dating back to 10,000 BC), neurosurgery still stands as one of the most dynamic fields within medicine today [8]. Given the wide variety in etiology, clinical presentation, and pathohistological features touted amongst the variety of potential intracranial and CNS tumors, neurosurgery continues to discover new avenues for greater treatment interventions and more efficient intraoperative analyses. Given that the current prevalence of said pathology has continued to remain primarily stagnant (with only marginal reductions in case prevalence since 2008), it is imperative that current clinicians remain vigilant in aiming to appreciate the medley of possible treatment approaches that are practically applicable in the world of neurosurgical techniques. This review specifically aims to discuss advancements in intraoperative histology in the realm of neurosurgery, highlighting the role of these innovations in improving patient outcomes [3,4].

## 2. Frozen Sectioning

**Figure 1:**
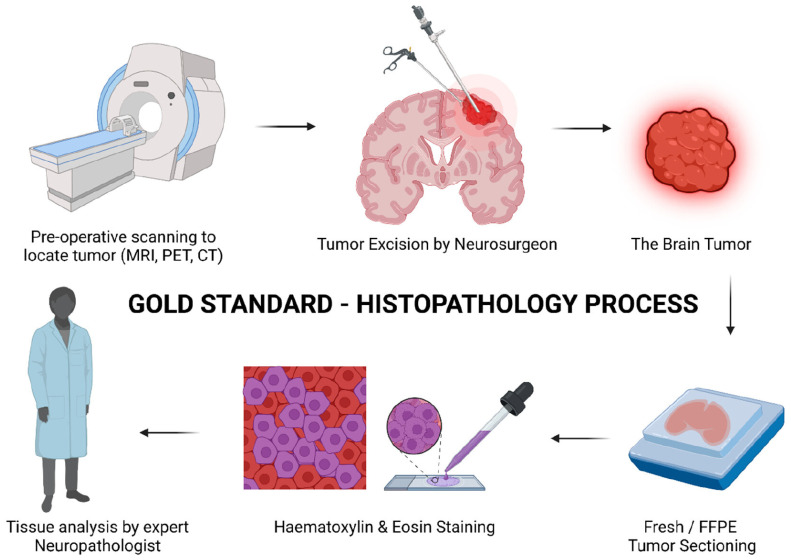
Schematic representation of the long and tedious histopathology process. The patients need to undergo initial scanning to identify the tumor. This is followed by a sample section which further undergoes multiple processing steps, taking a longer time, and needs to be confirmed by a neuropathologist which increases the chances of error. An error or partial removal of a tumor leads to invasive surgery, again affecting the health and quality of life of the patient (created with Biorender.com). Copyright © 2023 The Authors. CS Omega published by American Chemical Society [13].

An intraoperative analysis of tissue samples is crucial to allow for a surgeon to determine established treatment protocols and margins in the neurosurgical setting. A proper analysis of tissue requires the hardening of the matrix to allow for sectioning. This led to the introduction of frozen sectioning into the field of intraoperative pathology (Figure 1). The first instance of this technique dates back to 1905 when Dr. Louis B. Wilson simply used cold winter air to help freeze his samples [9]. Dr. Wilson then progressed to using the CO_2_ microtome and staining samples with methylene blue before many other advancements were made to the current state of frozen cytology [10]. The most current form of frozen sectioning includes the utilization of a cryostat set between −20 and −30 degrees Celsius and frozen aerosol sprays to help set the sample [11]. A study by Novis and Zarbo found that 90% of frozen sectioning block turnaround times were within 20 min from the time the pathologist received the sample [12]. This fast turnaround time has allowed for the expansion of an intraoperative diagnosis of lesions, rather than waiting for a traditional pathology report after the operation has been completed. 

Frozen sectioning has been proven to be a fast intraoperative process, yet many individuals have questioned the accuracy of such procedures. A study performed by Khoddami et al. (2015) found an accuracy rate of 99.5% for the frozen sectioned samples compared to the gold standard of formalin-fixed and paraffin-embedded samples [14]. The same article reported a sensitivity and specificity of 91.4% and 99.7%, respectively. The high level of accuracy associated with frozen sectioning was repeated in a study performed by Kang et al. (2019), which displayed an intraoperative accuracy of 98.8% compared to permanent histological diagnosis [15]. Another study demonstrated a high correlation of 95.6% between the intraoperative diagnosis and the final diagnosis [16]. These results demonstrated a high level of accuracy of intraoperative frozen sectioning, but several limitations must be considered while utilizing the technique.

The high level of accuracy of frozen sectioning does not make it a viable replacement for paraffin-embedded tissue techniques, which is an important distinction to make. One reason that it cannot be used as a replacement is the possibility of interpretative errors in frozen sections due to the freezing process distorting the architecture [17]. This interpretative error occurs commonly in differentiating cells that are spindle-shaped. This includes differentiating between cerebellopontine angle meningiomas and schwannomas [18]. Also, frozen sectioning provides the possibility that oligodendroglioma is mischaracterized as a high-grade astrocytoma. The perinuclear halo traditionally seen with paraffin-embedded oligodendroglia-based cells is lost in the freezing process [19]. The loss of a perinuclear halo along with freezing artifacts causes oligodendroglia to be angulated similar to astrocyte cells. Errors with frozen sectioning extend beyond these issues listed, but it is a valuable tool that can still provide accurate information. Proper anatomical knowledge, good surgical technique, and the expert pathological interpretation of neural tissue can help to reduce errors in differentiation between cell lines and structures [20]. 

Overall, frozen sectioning is a valuable intraoperative tool to help provide guidance to the surgeon on margins, diagnosis, and possible complications. Limitations exist in the usage of frozen sectioning due to the possibility of artifact and tissue distortion created during the freezing process. However, close cooperation between the surgeon and the pathologist can help to limit errors. The pathologist should have the opportunity to look at radiologic images before the procedure to be able to compare histological findings to what is seen in imaging modalities. The combination of radiologic images and histology has been shown to improve accuracy and avoid interpretative errors with frozen sectioning [11]. Continuing, the proper selection of tumors for the procedure improves accuracy. Masses firmer in consistency, such as fibroblastic meningioma, showed better yields from frozen cytology than more friable masses [21]. Lastly, the usage of intraoperative frozen cytology along with other diagnostic techniques such as crush smear has been shown to improve diagnostic accuracy [22]. In conclusion, the proper usage of frozen cytology has been shown to be a valuable tool in the belt of neurosurgeons to provide greater guidance for intraoperative decision-making.

## 3. Other Cytological Preparations

Many other cytological techniques can aid in intraoperative diagnosis and care during neurosurgical procedures. These techniques include crush smear, touch imprint, and squash techniques. These techniques help to fill in the gaps provided by frozen sectioning, including time constraints, specialized personnel, and cost. The smear technique can originate with a small tissue sample taken through a burr hole. The tissue sample can then be crushed between two slides utilizing the soft, gel-like consistency of central nervous system tumors [23]. This technique can be performed in 10–20 min without the need for special equipment, which makes the technique faster and easier to perform than frozen sectioning [24]. As no extra equipment is utilized, smears tend to be a more cost effective technique than frozen sectioning. Smear techniques have also shown a fair degree of accuracy in diagnosis. A study by Govindaram et al. (2017) demonstrated a diagnostic accuracy of 90.67% with a sensitivity of 98.7% [25]. Therefore, smear provides a valuable diagnostic tool which can be used in resource-poor areas to aid in intraoperative diagnosis.

Touch imprint cytology is another useful technique that can aid in intraoperative diagnosis if used with the correct indications. This technique utilizes a sample from the lesion and contacts, and then it removes the sample from a glass slide [26]. This leaves an imprint in the form of the sample on the slide, which is then stained commonly with hematoxylin and eosin. This process has been shown to be very effective for the analysis of pituitary neuroendocrine tumors where it can be difficult to produce wide tumor margins [27]. The technique has been highly effective for primary central nervous system tumor grading with an accuracy rate of 94.6%. However, the success rates have staggered in metastatic tumors with a rate of only 61.5% [26]. This discrepancy highlights the need for a vast array of intraoperative cytological techniques in the field of neurosurgery. Proper knowledge of indications, strengths, and weaknesses can aid a care provider in determining which technique will provide the most diagnostic value for each individual case.

## 4. Raman Histology 

In recent years, the development of various intraoperative neurohistological analyses has emerged based on advancements in the application of Raman spectroscopy. The Raman scattering phenomenon was first observed in 1928 by Sir Chandrasekhara Raman, leading to the development of the first Raman spectroscopy microscopes in the 1970s [28]. The Raman scattering phenomenon posits that the spontaneous inelastic scattering of light will occur based on the molecular characteristics of the sample [29,30]. Therefore, Raman spectroscopy allows for the rapid, non-destructive acquisition of label-free information directly from the sample’s chemical characteristics [7,29]. As most neuropathologies involve alterations at the molecular level, Raman technology has been increasingly efficacious in rapidly diagnosing and treating ischemic or traumatic brain injuries, neurodegenerative diseases, and brain tumors [29]. Several techniques for enhancing Raman spectroscopy have been developed, as spontaneous Raman spectroscopy alone is not strong enough to provide high-resolution images. These methods include, but are not limited to, Stimulated Raman Histology (SRH), Coherent Anti-Stokes Raman Scattering (CARS), Surface-Enhanced Raman Scattering (SERS), and Tip-Enhanced Raman Scattering (TERS) [31]. For instance, compared to conventional methods, Stimulated Raman Histology (SRH) microscopy allows for the rapid and accurate detection of the extent of brain tumor infiltration through high-resolution imaging [32]. A review of the existing literature indicates that as Raman histological techniques continue to develop and improve, they will become an essential element in the neurosurgeon’s armamentarium for accurate intraoperative diagnosis and decision-making [33].

### 4.1. Indications

Raman technology has very promising indications in oncological neurosurgery, as it allows for the detection of microscopic cancer cell infiltration and therefore more accurate resection of brain tumors and diagnosis of tumor subtypes [34]. Perhaps the primary advantage of Raman spectroscopy is its ability to rapidly produce histological images, allowing the neurosurgeon to achieve empirically clear tumor margins and enhancing the prognosis of neuro-oncologic patients [34]. Raman-based imaging methods can also be used in the detection of neurodegenerative diseases such as Alzheimer’s disease (AD). Raman spectroscopy has been shown to greatly improve the accuracy of diagnosing such disorders by detecting the presence of neurofibrillary tangles, amyloid-β plaques, and tau protein in tissues [35]. A study by Ryzhikova et al. suggests that an RS analysis of cerebrospinal fluid may allow for the early detection of Alzheimer’s, as they were able to diagnose AD with 84% specificity and sensitivity [35,36]. Another study by Lochoki et al. successfully used RA to detect amyloid-beta peptides in tissue samples from AD patients [35,37].

Raman-based imaging techniques may also be effective in analyzing ischemic metabolic changes in stroke and TBI patients. For example, the release of cytochrome C from damaged neurons can be measured by RS to elucidate the extent of ischemic cell death [29].

### 4.2. Techniques

#### 4.2.1. Raman Spectroscopy

Raman spectroscopy (RS) has promising clinical applications in the detection of healthy versus necrotic or diseased brain tissue as well as the detection of biomarkers that may indicate the stage of the pathology [35]. Raman imaging uses monochromatic light that interacts with the vibrational modes of a sample, causing an inelastic scattering of photons. The Raman phenomenon can be analyzed through Stokes or anti-Stokes scattering, where the scattered beams have a lower or higher frequency than that of the incident beam, respectively [35,38]. The resultant peaks represent the Raman spectrum, and the energy shift is measured as the “Raman shift”, which indicates the various concentrations of specific molecules based on the strength of each spectroscopic peak. This allows each protein, lipid, and DNA molecule to be distinguished through its unique vibrational fingerprint [35]. However, due to the relatively weak signal given by spontaneous Raman spectroscopy, various techniques have been developed to amplify the signal, including Stimulated Raman Scattering (SRS) Spectroscopy, Coherent Anti-Stokes Raman Scattering (CARS) Microscopy, Surface-Enhanced Raman Scattering (SERS) Microscopy, and Tip-Enhanced Raman Scattering (TERS) microscopy [31,35]. Furthermore, the invention of the femtosecond laser has allowed for the development of other SRS techniques with minimal collateral tissue damage, as its pulse duration is a minuscule 10^−15^ s [39].

#### 4.2.2. Stimulated Raman Scattering (SRS) Microscopy

Stimulated Raman Scattering (SRS) microscopy utilizes two lasers, the pump beam, and the Stokes beam, to amplify the Raman signal given by specific bonds in the sample and generate image contrast [40]. Inverted microscopes with two lenses are usually used for SRS; the first lens projects beams onto the sample and the second funnels the photons into the detector [30]. The Stokes beam is typically a fixed wavelength while the Pump beam is a tunable wavelength and both pulse at a rate of 80 MHz. The Stokes and Pump beams must overlap for SRS to occur; this is achieved through the microscope’s dichroic mirror and optical delay line [41]. The Stokes beam is then reduced to a fixed frequency of 1-20 MHz using an optic modulator and optic filters to elucidate the SRS signal [30,41]. A large-area photodiode detector and optical filters are then used to retrieve the unmodulated beam from the overlapping Stokes and Pump beams, and the signal is finally converted to a histological image through specialized computer software [41].

Currently, SRS microscopy devices such as Invenio Imaging Inc.’s NIO Laser Imaging System are emerging as valuable tools in the operating room. The NIO Laser Imaging System harnesses Stimulated Raman Histology (SRH) and laser spectroscopy to allow for rapid intraoperative histologic evaluation, producing digital hematoxylin and eosin (H&E)-stained slides and optimizing the localization of brain tumor infiltration [7]. The NIO is therefore particularly useful for maximizing the complete resection of tumor margins and improving outcomes in neuro-oncological patients [42].

#### 4.2.3. Coherent Anti-Strokes Raman Scattering (CARS)

Coherent Anti-Stokes Raman Scattering (CARS) microscopy is one method that was developed to overcome the imaging speed limitations of spontaneous Raman scattering [43]. As in SRS, the Pump and Stokes beams concurrently excite the sample such that the frequency difference between the beams corresponds to the vibrational frequency of a specific chemical bond in the sample. However, in addition to stimulated Raman gain (ω_s_) and loss (ω_p_), CARS produces two much stronger signals: Coherent Anti-Stokes Raman Scattering ((ω_p_−ω_s_) + ω_p_) and Coherent Stokes Raman scattering (ω_p_−(ω_p_−ωs)) frequencies [43]. For this reason, CARS is significantly more sensitive than spontaneous Raman spectroscopy and allows for extremely rapid imaging [44]. Furthermore, the nonlinear nature of CARS allows for 3D sectioning of thick tissues, and tissue damage is minimized as CARS occurs in the ground electric state [44].

#### 4.2.4. Surface-Enhanced Raman Scattering (SERS) Microscopy

Another method of enhancing the sensitivity of spontaneous Raman scattering is Surface-Enhanced Raman Scattering (SERS) microscopy [41]. The SERS method was discovered serendipitously when it was observed that a roughened silver electrode significantly increased the Raman signal of an adsorbed pyridine sample [45]. SERS utilizes a metal probe to concentrate electromagnetic energy through surface plasmons, therefore significantly enhancing the scattering of photons [46,47]. The probe must be composed of a transition metal, a noble metal, or a semiconductor to allow for molecules to be rapidly analyzed on the surface of the metal [47]. The use of such metal probes enhances the incident light and therefore increases Raman scattering, providing a faster and clearer image [41].

#### 4.2.5. Tip-Enhanced Raman Scattering (TERS) Microscopy

Tip-Enhanced Raman Spectroscopy (TERS) operates based on the chemical sensitivity of the SERS method, with the added benefit of high spatial resolution at the sub-nanometer level via scanning probe microscopy (SPM) [48]. Like SRS, CARS, and SERS, TERS is rapid, accurate, and non-destructive. The TERS method involves a sharp metal SPM tip with an enhanced electromagnetic field at the apex due to the resonance of surface plasmons, thereby significantly augmenting the Raman signal of molecules in its vicinity [48]. This localization of the signal at the probe tip allows TERS to overcome the diffraction limit of SRS and SERS and convey improved sub-diffraction limit spatial resolution [49].

### 4.3. Outcomes

Several ex vivo studies have found Raman spectroscopy (RS) to be highly specific and highly sensitive, with >90% accuracy in distinguishing neoplastic from normal tissue [50]. Zhang et al. reported the detection of brain tumors by RS to be 97% sensitive and 98.5% specific [50,51]. Another ex vivo study by Aguiar et al. resulted in 97.4% sensitivity and 100% specificity in distinguishing meningiomas, medulloblastomas, and glioblastomas from healthy tissue [50,52]. These highly promising ex vivo results have laid the foundation for the in vivo use of Raman spectroscopy. In a study of 13 glioma patients, RS was used to detect invasive cancer cells that would not be detected with T1 or T2 MRI. It was found that RS was able to intraoperatively detect residual grade 2–4 gliomas with 93% sensitivity and 91% specificity [53].

In 2012, Freudiger et al. developed Multicolored Coherent Raman Imaging (MC-CRI), an advanced imaging technique that combines the principles of SRS and CARS microscopy with multiple wavelengths of excitation light to enable the concurrent visualization of various molecular species within the sample. They used this technology to identify lipid and protein signals from CH2 and CH3 vibrations in brain tissue ex vivo, allowing them to identify various central nervous system pathologies in mouse models [54].

In 2017, Orringer et al. described the first application of intraoperative stimulated Raman scattering (SRS) microscopy and developed the stimulated Raman histology (SRH) interpretation technique, which utilizes SRS images to produce simulated hematoxylin and eosin (H&E) stains [7]. They found that in a sample of 30 patients, the intraoperative use of SRH has over 92% accuracy when compared to conventional histological methods in diagnosing brain tumors, and 90% accuracy in elucidating tumor subtypes. In another study on the efficacy of SRH, they created a residual network to predict the presence of tumor cells, non-tumor cells, and low-quality images. Compared to the neuropathologist’s analysis of the samples, the SRH-based residual network predicted the presence of tumor cells with 90.2% accuracy in under four minutes [7].

The use of Coherent Anti-Stokes Raman scattering (CARS) has also illustrated promising results by significantly decreasing the time required for image collection. In a landmark study by Evans et al., the speed of generating CARS images was found to be 30 images per second, compared to a 30 min CARS image collection time in 1999 [55]. This reduction in image generation time greatly enhances intraoperative decision-making as well as patient prognoses.

Furthermore, Surface-Enhanced Raman Scattering (SERS) microscopy has been shown to significantly improve prognosis in post-resection glioma. Han et al. used a SERS scanner intraoperatively until all Raman signals were completely absent in the tissue, and longitudinal MRI analysis indicated that this SERS-guided resection greatly reduced glioma recurrence rate in rat models. SERS has also been used to distinguish acidic margins of gliomas, as extracellular acidosis is a marker for cancer cell infiltration [56]. Jin et al. used a SERS navigation system to locate these acidic margins in animal models as well as in excised tissue from glioma patients and found that the level of acidity correlates with the level of cancer cell proliferation and density. The post-operative survival of animal models was greatly enhanced compared to conventional methods, indicating a promising prognosis for glioma patients [56].

Finally, Tip-Enhanced Raman Scattering (TERS) microscopy achieves both the enhancement of SERS and the high spatial resolution of SPM, thereby overcoming the diffraction limit for more accurate images [57]. As TERS is a relatively new technique and faces limitations in size and shape, the literature shows limited in vivo analysis of SERS in neurosurgery.

## 5. Confocal Laser Endomicroscopy

**Figure 2:**
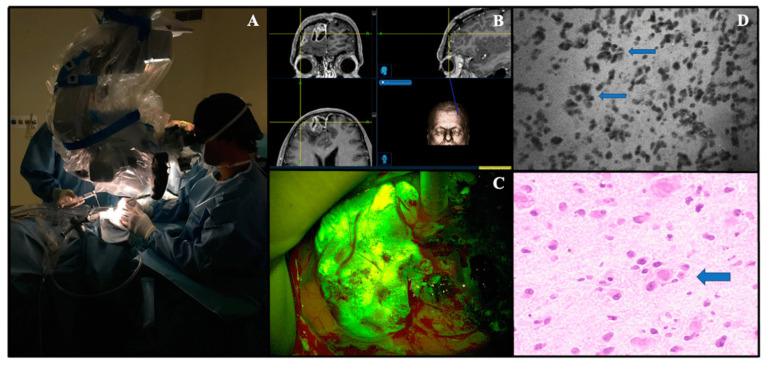
Confocal Laser Microscopy in vivo Convivo case (Besta Neurological Institute, Milan, Italy). (**A**). The confocal probe is dressed with its appropriate sterile sheath and used directly on the cerebral surface. (**B**). Preoperative magnetic resonance with contrast administration images loaded on the neuronavigation system (Stealth S8-Medtronic) of a right frontal parasagittal anaplastic oligodendroglioma, IDH mutant (WHO grade III). (**C**). Intraoperative view of fluorescein-guided removal of the tumor under YELLOW560 filter activation on a Pentero microscope (Carl Zeiss Meditec). (**D**). Convivo in vivo image taken at the center of the tumor showing tumor cells along with typical perineural satellitosis (small arrows), which can be easily found on a relative histopathological image as well (hematoxylin and eosin, big arrow, (**E**)). Copyright© 2021 The Authors. Journal of Clinical Medicine published by MDPI, Basel, Switzerland [60].

Confocal Laser Endomicroscopy (CLE) has grown in popularity for intraoperative, real-time imaging in the operating room (Figure 2). Real-time imaging is particularly useful in tumor resections as surgeons can effectively evaluate histoarchitecture and the need for the resection of specific pieces of tissue. Using intraoperative imaging reduces the time required for tissue collection and standard biopsy procedures involving in-person pathologists. Instead, surgeons can employ the CLE in the operating room and send the live camera feed to a pathologist remotely or in person for analysis. With a pathologist on alert, an analysis of histoarchitecture can be performed instantly. As such, the surgeon can more effectively execute the resection [58]. CLE has been widely implemented in gastroenterology because of its remarkable accuracy in detecting neoplasia of the colon [59]. However, the technology has yet to reach widespread practice in neurosurgery, and the gold standard remains frozen sectioning. 

### 5.1. Indications

Because CLE allows surgeons an insight into the histological and cytologic features of tissues, most indications are neoplastic in nature. It allows surgeons to determine if a tissue is dysplastic, neoplastic, or healthy. More specifically, CLE is used to investigate, monitor, and resect tissue samples from meningiomas, gliomas, and pituitary adenomas. 

### 5.2. Techniques

CLE devices are classified as either probe-based or endoscope-based. Regardless, the laser is directed until it is in direct contact with the tissue of interest. The laser then delivers a straight line of light into the tissue. Light rays reflect off the tissue back towards the laser, and reflections are redirected through the same pinhole as the light source (thus the name confocal). The sensor is placed proximally to the pinhole, excluding all the scattered or refocused light from the imaging plane. By doing so, the apparatus significantly increases the spatial resolution of the image [61,62]. An additional measure to increase resolution has been the advent of a photoactive contrast enhancer, the contrast typically being fluorescein sodium [63,64].

It should be noted that Confocal Reflectance Endomicroscopy (CRE) is a very similar technique to Confocal Laser Endomicroscopy (CLE), although they differ in their light source and applications. CLE uses a coherent laser as its light source to excite fluorescent molecules and create an image of cellular structures, such as tumor cells. Instead, CRE uses non-coherent light such as halogen lamps or light-emitting diodes (LEDs), and reflected light from the surface of the sample forms images of surface morphology rather than internal structures [65]. Although the use of CRE alone in neurosurgery is feasible, the current literature focuses on the application of CLE and potentially multimodal confocal microscopes that combine the two. These multimodal devices harness both the histopathological report from CLE and information on the specimen’s surface morphology and vascularity from CRE [66].

### 5.3. Outcomes

Image quality, brightness, contrast, and resolution are important parameters to examine. Brightness and contrast have been shown to be higher for in vivo imaging that uses CLE relative to ex vivo images that use traditional biopsy methods [63,67]. Another non-clinical measure critical to CLE is the reduction in time spent on tissue analysis. In 2016, Martirosyan et al. conducted one of the first preclinical tests for feasibility. The study showed a decrease in time as the system was only in use for an average of 15.7 min, a marked improvement from the typical 20+ minutes required for frozen histology [68]. 

Feasibility of CLE was further confirmed by determining comparable, if not better, accuracy with decreased time spent on analysis [69,70,71]. First, CLE was compared to frozen histology in ex vivo studies. In doing so, concordance was found to be 80% for determining correct diagnosis and 93.3% for categorizing patterns at the tumor core [72].

Only a handful of studies have been performed to show the use of CLE in vivo. In the first, intraoperative CLE was shown to correctly diagnose neoplasia while reducing operation time. The device was reported as easy to use due to its similarity to other microsurgical instruments [73].

A later study showed, in a 30-patient sample, that CLE could be used with nearly identical accuracy to frozen sectioning and permanent histology. The diagnostic accuracy, sensitivity, and specificity reported for CLE vs. the frozen section were 94%, 94%, and 100%; and for CLE vs. permanent histology, they were 92%, 90%, and 94%, respectively [74].

## 6. Conclusions

The field of neurosurgery faces ongoing challenges in the diagnosis and treatment of CNS malignancies. Intraoperative histology has emerged as a critical tool in guiding surgical decision-making, with techniques such as frozen sectioning, Raman histology, and Confocal Laser Endomicroscopy (CLE) offering invaluable insights into tumor pathology. Frozen sectioning provides rapid intraoperative analysis in tumor resection, while highly accurate, tissue distortion and interpretive errors remain challenges. Stimulated Raman Histology (SRH) offers high-resolution imaging and has demonstrated efficacy in the diagnosis of brain tumors and their subtypes. Surface-Enhanced Raman Scattering (SERS) microscopy has shown promise in detecting tumor margins and reducing glioma recurrence rates, and Tip-Enhanced Raman Scattering (TERS) microscopy conveys enhanced spatial resolution. Coherent Anti-Stokes Raman Scattering (CARS) microscopy allows for rapid imaging and improved intraoperative decision-making, and Confocal Laser Endomicroscopy (CLE) enables the real-time visualization of histoarchitecture and therefore precise tumor resection. The incorporation of these intraoperative histological techniques into clinical practice holds great potential for improving patient outcomes in neurosurgical oncology. The continued investigation and refinement of these methods are essential to further advance their utility and address existing limitations. By harnessing the power of intraoperative histology, neurosurgeons can strive towards more precise and personalized treatments for patients with CNS malignancies.

## Data Availability

Not applicable.
